# Genetic polymorphisms of cell adhesion molecules in Behcet’s disease in a Chinese Han population

**DOI:** 10.1038/srep24974

**Published:** 2016-04-25

**Authors:** Minming Zheng, Lijun Zhang, Hongsong Yu, Jiayue Hu, Qingfeng Cao, Guo Huang, Yang Huang, Gangxiang Yuan, Aize Kijlstra, Peizeng Yang

**Affiliations:** 1 The First Affiliated Hospital of Chongqing Medical University, Chongqing Key Laboratory of Ophthalmology and Chongqing Eye Institute, Chongqing, P. R. China; 2 University Eye Clinic Maastricht, Maastricht, The Netherlands

## Abstract

Cell adhesion molecules (CAMs) are involved in various immune-mediated diseases. This study was conducted to investigate the association of single nucleotide polymorphisms (SNPs) of CAMs with Behçet’s disease (BD) in a Chinese Han population. A two-stage association study was carried out in 1149 BD patients and 2107 normal controls. Genotyping of 43 SNPs was performed using MassARRAY System (Sequenom), polymerase chain reaction-restriction fragment length polymorphism (PCR-RFLP) and TaqMan SNP assays. The expression of CD6 and CD11c was examined by real-time PCR and cytokine production was measured by ELISA. A significantly higher frequency of the CT genotype, and a lower frequency of the CC genotype and C allele of CD6 rs11230563 were observed in BD as compared with controls. Analysis of CD11c rs2929 showed that patients with BD had a significantly higher frequency of the GG genotype and G allele, and a lower frequency of the AG genotype as compared with controls. Functional experiments showed an increased CD11c expression and increased production of TNF-α and IL-1beta by LPS stimulated PBMCs in GG carriers of CD11c rs2929 compared to AA/AG carriers. Our study provides evidence that CD6 and CD11c are involved in the susceptibility to BD in a Chinese Han population.

Uveitis is a vision-threatening intraocular inflammatory disease and a major cause of visual impairment and blindness. Behçet’s disease (BD) has been shown as the most common sight threatening uveitis entity in China^1^. BD is a recurrent systemic inflammatory disease characterized by major symptoms such as orogenital ulcers, skin lesions, and intraocular inflammation^2^. As yet, the pathogenesis of BD remains unclear. BD patients are currently treated with various immunosuppressive agents, but unraveling of the inflammatory pathways could lead to a tailored specific therapeutic approach that may prevent the blinding complications of the disease.

Migration of cells to the site of inflammation is a key event during uveitis and has been investigated previously in both animal models^3^ and in clinical uveitis^4^. Most of these studies have examined the role of CAMs protein expression in BD^5^. A thorough immunogenetic approach of CAMs in this disease has not yet been addressed and was therefore the subject of the study presented here.

Cell adhesion molecules (CAMs) are a group of proteins involved in cell binding or interaction between extracellular matrix (ECM) and cells. CAMs have been classified into four protein families: Ig (immunoglobulin) superfamily (IgSF, CAMs), the integrins (ITGA, IGTB), the cadherins (CDH), the selectins, and other uncategorized molecules. At an early stage of inflammation, leukocytes first adhere to the activated endothelium, and then infiltrate into the vessel wall, in association with an increasing expression of CAMs^6^. Various studies have focused on protein expression of CAMs in patients with inflammatory and autoimmune diseases^7^. Blocking ICAM-1 has been shown to significantly weaken the migration of Th1- and Th17-polarized cells^8^.

An abundance of gene association studies with CAMs, including ICAM1^9^,^10^,^11^, ICAM3^11^, ICAM5^12^, ITGAV^13^,^14^,^15^, ITGB^16^, LAMB1^17^, ALCAM^18^, CDH1^19^,^20^, CDH23^21^, CDHR3^22^, ITGAM^23^, selectins^24^,^25^,^26^, CD6^27^,^28^, CD11a^29^, CD11c^29^, CD18^29^, CD28^30^, CD44^31^, CD48^28^, CD58^11^,^30^, CD80^31^,^32^,^33^, CD86^11^, CD103^34^, and CD226^28^,^35^ have recently been reported for several inflammatory or autoimmune diseases. Although some studies have addressed the association of selected CAMs with uveitis^10^,^29^, no reports are available concerning the association between genetic polymorphisms of the complete family of cell adhesion molecules with ocular BD. In this study we therefore investigated whether genetic variants of cell adhesion molecules may confer susceptibility to BD in a Chinese Han population. We identified two genetic loci, rs11230563 in CD6 and rs2929 in CD11c, that contribute to the risk of BD.

## Results

### Clinical characteristics of patients with BD

The clinical characteristics, gender and age of the enrolled BD patients and controls are displayed in Table 1. The genotype frequencies of the 43 SNPs did not deviate from the Hardy–Weinberg equilibrium in the controls.

### Allele and genotype frequencies of tested SNPs in patients and controls in the first stage study

A total of 391 BD patients and 603 healthy controls were enrolled in the first stage study. An increased frequency of the rs2929 GG genotype in CD11c was observed in patients with BD (Pc = 0.034, OR = 1.69) (Table 2). The frequency of the AG genotype of rs2929 in patients with BD was significantly lower than that in normal controls (Pc = 0.019, OR = 0.56) (Table 2). In the case of rs11230563 in CD6, an increased frequency of the CT genotype was observed in BD patients (Pc = 8.624 × 10^−4^, OR = 1.94), whereas a decreased frequency of the C allele and CC genotype (Pc = 1.371 × 10^−3^, OR = 0.59; Pc = 7.380 × 10^−4^, OR = 0.52, respectively) was found (Table 2). We were not able to detect a significant association between the other 41 SNPs investigated and risk of acquiring BD (Supplementary Table S1).

### Allele and genotype frequencies of tested SNPs in patients and controls in the second stage study and combined study

To further verify the observed association of CD6 and CD11c with BD, we replicated the associated SNPs rs2929 and rs11230563 using a different cohort of patients that included 758 cases and 1504 controls. The results showed that the frequencies of the rs2929/CD11c GG genotype and G allele were significantly higher in BD patients (Pc = 1.881 × 10^−4^, OR = 1.51; Pc = 7.808 × 10^−5^, OR = 1.44, respectively), and lower frequencies of the AG genotype (Pc = 1.184 × 10^−3^, OR = 0.69) when compared with controls (Table 2). As for rs11230563/CD6, decreased frequencies of the C allele and CC genotype (Pc = 2.352 × 10^−5^, OR = 0.66; Pc = 1.087 × 10^−5^, OR = 0.63, respectively) were found, whereas an increased frequency of the CT genotype was observed (Pc = 1.628 × 10^−4^, OR = 1.52) (Table 2). Combining the data from the first and second stage study showed that rs2929 in CD11c was associated with the susceptibility to BD (GG genotype: Pc = 5.573 × 10^−6^, OR = 1.56; AG genotype: Pc = 2.087 × 10^−5^, OR = 0.64; G allele: Pc = 1.398 × 10^−5^, OR = 1.45) (Table 2), and that rs11230563 in CD6 also conferred susceptibility to BD (CC genotype: Pc = 1.097 × 10^−8^, OR = 0.60; CT genotype: Pc = 3.266 × 10^−7^, OR = 1.63; C allele: Pc = 7.594 × 10^−9^, OR = 0.64) (Table 2).

Because the gender distribution of patient and control groups was different, we also calculated genotype and allele distribution of rs11230563 and rs2929 according to gender in controls and patients separately. The result showed that the genotype and allele frequencies of rs2929 showed significant differences between patients and controls in both male (G allele: Pc = 5.034 × 10^−4^, OR = 1.37; AG genotype: Pc = 1.289 × 10^−3^, OR = 0.69; GG genotype: Pc = 6.102 × 10^−4^, OR = 1.46) and female patients (G allele: Pc = 0.002, OR = 1.69; AG genotype: Pc = 0.012, OR = 0.57; GG genotype: Pc = 0.006, OR = 1.84). Likewise, the genotype and allele frequencies of rs11230563 also showed significant differences between patients and controls in both male (C allele: Pc = 2.290 × 10^−7^, OR = 0.64; CT genotype: Pc = 1.886 × 10^−6^, OR = 1.66; CC genotype: Pc = 2.480 × 10^−7^, OR = 0.59) and female patients (C allele: Pc = 0.002, OR = 0.63; CC genotype: Pc = 0.012, OR = 0.60) (Table 3). We subsequently explored whether the association with rs2929 and rs11230563 behaved as dominant or recessive using univariate and multivariate logistic regression analysis. The CD11c-rs2929 A allele association with BD behaved as a dominant model (Table 4). The CD6-rs11230563 T allele association with BD behaved as both dominant and recessive (Table 5).

### Stratified analysis of rs2929 and rs11230563 with main clinical features of Behcet’s disease

A stratified analysis was carried out to examine the association of rs2929 and rs11230563 with the main clinical features of BD. The main clinical features of BD included genital ulcer, skin lesions, arthritis, positive pathergy reaction and hypopyon. No significant association was found for the individual extraocular manifestations of BD with the tested SNPs (Supplementary Tables S2 and S3).

### The influence of rs11230563 on CD6 and rs2929 on CD11c expression

**Figure 1:**
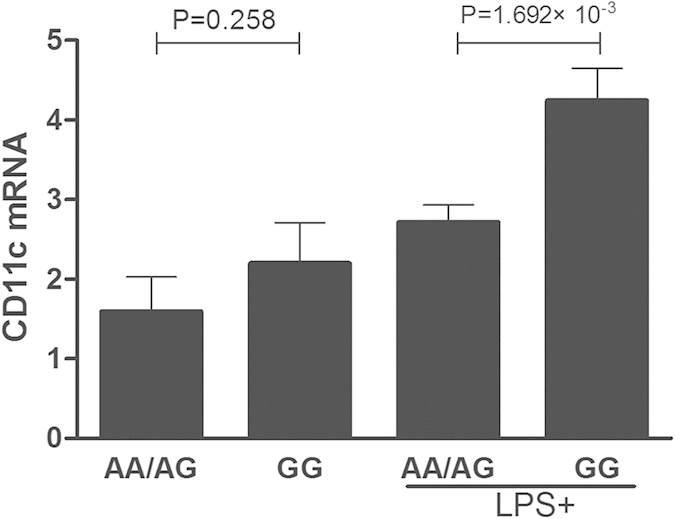
The influence of various rs2929 genotypes on the expression of CD11c. CD11c expression in non-stimulated PBMCs and LPS stimulated PBMCs from normal controls carrying different genotypes of rs2929 (AA/AG = 16, GG = 16).

The aforementioned results revealed that genetic polymorphisms of CD6 and CD11c are associated with susceptibility to BD. We subsequently investigated mRNA expression of CD6 and CD11c in PBMCs from 32 healthy individuals with a known genotype. No significant association was found in CD11c expression between the various genotypes of rs2929 when PBMCs were left unstimulated (Fig. 1). However, following stimulation with LPS, the mRNA expression of CD11c rs2929 in GG cases was significantly increased as compared to AA/AG carriers (P = 1.692 × 10^−3^) (Fig. 1). Different genotypes of rs11230563 did not affect CD6 mRNA expression levels regardless whether PBMCs had been stimulated with LPS or not (Supplementary Figure 1).

### The influence of rs2929 on cytokine production

**Figure 2:**
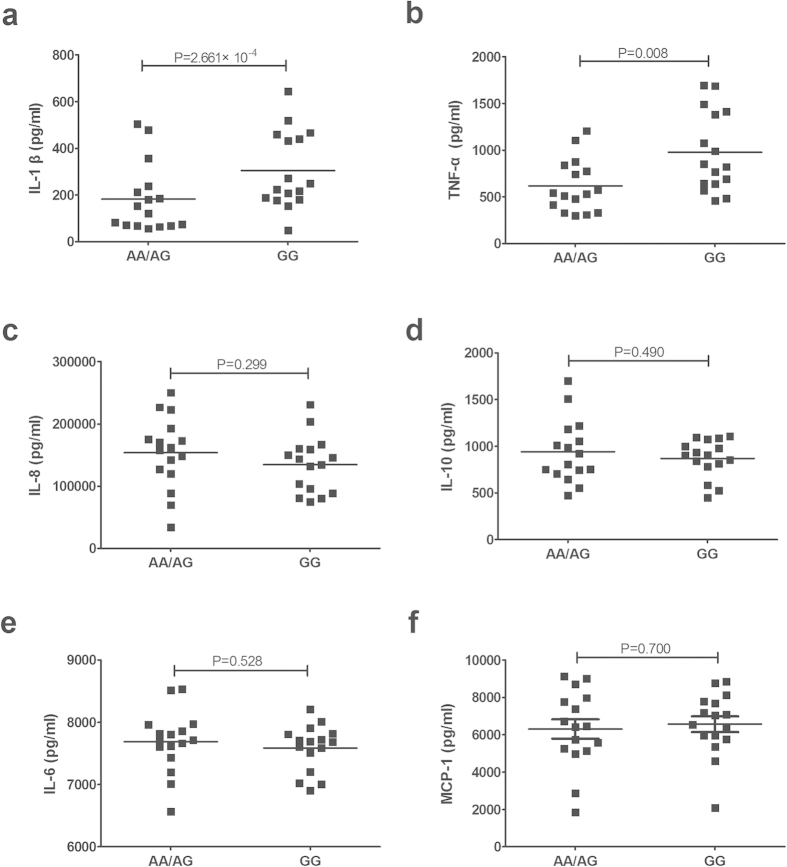
The influence of rs2929 on cytokine production. The production of IL-1beta (**a**), TNF-a (**b**), IL-8 (**c**), IL-10 (**d**), IL-6 (**e**) and MCP-1 (**f**) by LPS stimulated PBMCs from normal controls carrying different genotypes of rs2929 (AA/AG = 16, GG = 16).

As we found that different genotypes of rs2929 could affect CD11c expression following LPS stimulation, further functional experiments were performed to investigate whether different genotypes of rs2929 could also affect the cytokine production of LPS stimulated PBMCs. This was performed using cells obtained from 32 healthy genotyped individuals. We chose to study IL-1beta, IL-6, IL-8, IL-10, TNF-α and MCP-1, cytokines that have been shown to play a crucial role in the pathogenesis of BD. IL-1beta production by stimulated PBMCs from GG carriers was higher than that seen in AA/AG (P = 2.661 × 10^−4^) carriers (Fig. 2a). Similarly, an increased TNF-α production by stimulated PBMCs was also observed in GG compared to AA/AG (P = 0.008) carriers (Fig. 2b). No effect of the various rs2929 genotypes on the release of the other four cytokines could be detected (Fig. 2c–f).

## Discussion

The role of genetic polymorphisms in genes coding for cell adhesion molecules in the risk of developing BD was investigated in this study and showed that rs2929 of CD11c and rs11230563 of CD6 were significantly associated with BD. Using functional studies we found that the relative mRNA expression levels of CD11c were increased in individuals with the GG genotype of rs2929. Additionally, IL-1beta and TNF-α production by PBMCs were significantly increased in individuals with the GG genotype of rs2929 as compared with the other two genotypes.

We were not able to find evidence for an association between genetic variants of ICAM1, CD11a, CD18 and BD susceptibility. Earlier studies performed by other groups demonstrated a significant association of rs5498, rs11574944, rs2230429, rs235326 with BD risk^10^,^29^. The lack of association in our study may be due to geographical, ethnical or clinical differences.

Although earlier studies showed evidence of an association with SNPs of CD11a, CD11c, CD18 in BD patients from Korea^29^ and with SNPs of ICAM1 in BD patients from Tunisia^10^, the genetic polymorphisms of cell adhesion molecules have not yet been reported for large cohorts of BD patients in Chinese Han. Our data are similar to the Korean study^29^ that showed that the frequencies of the CD11c rs2929 GG genotype and G allele were significantly higher in BD patients than in controls. The odds ratio for the G allele association with BD was 1.4 in the Korean study and in our study it was 1.46. The G allele of rs2929 is a common variant in both Chinese Han and in Koreans with a frequency of 86% and 67%, respectively. In the Korean study, the patients were recruited at a department of dermatology and 61% of patients exhibited ocular symptoms. The fact that a similar association was seen with rs2929 whether patients had uveitis or not suggests that the association is not confined to those with ocular disease, which is also in agreement with our findings showing no difference between our different BD subgroups. On the other hand, the Korean study did note that the CD11c GG genotype of rs2929 was significantly more frequent in the patients with arthritis than in those without (66.3% versus 49.1%) as well as in patients with neurologic involvement compared to those without (10.0% versus 55.9%). We were not able to confirm the association found in Korean BD patients for the C allele of rs2230429 in the CD11c (OR = 1.7), which may be due to a difference in the patient population studied. An association of the CD11c rs2929 A allele with gastric ulcers has been reported for Caucasians^36^ whereas earlier studies from this group could not provide evidence for an association with inflammatory bowel disease^37^. Why gastric ulcers are associated with the A allele and BD is associated with the C allele is not clear.

The CD11 gene cluster maps on chromosome 16p11–12 and includes the integrin alpha L (CD11a), integrin alpha M (CD11b), integrin alpha X (CD11c), and integrin alpha D (CD11d) chain^36^. These integrin alpha chains form a heterodimeric molecule with the same integrin beta chain (CD18) and constitute a family of integral membrane glycoproteins expressed on leukocytes that play an essential role in the migration of white blood cells to the site of inflammation. Our observation that rs2929 G allele carriers show an enhanced expression of these adhesion molecules is in line with the observation that BD is a disease with an exaggerated neutrophil response leading to a vasculitis in many organs and tissues^38^.

The association we described between the CD6 SNP rs11230563 has not yet been reported earlier in BD, although a haplotype of CD6 containing rs11230563 and rs2074225 was shown to be associated with MS^39^. A recent study found that the rs12360861 in CD6 was associated with MS, although functional studies were not able to show that these CD6 SNPs affected its mRNA levels^40^. Studies in Caucasians demonstrated a significant association of the G allele of rs17824933 in CD6 with MS risk^12^, although this could not be confirmed in Korean patients^24^. The MS risk allele in the rs17824933 CD6 locus may result in an alternative splicing of the gene^41^ and this locus has been found to affect proliferation of CD4 (+) T cells^42^.

CD6 is a membrane glycoprotein, which is mainly expressed on T cells. It interacts with its ligand activated leukocyte cell adhesion molecule (ALCAM/CD166), which is expressed on many cell types including nervous system cells, epithelial cells, mesenchymal cells, endothelial cells and leukocytes^43^. The CD6 ALCAM interaction plays an important role in the immune response^44^ and has been implicated in autoimmune diseases such as multiple sclerosis^27^, rheumatoid arthritis^45^ and psoriasis^46^. A humanized monoclonal antibody directed against CD6, named Itoluzimab has recently been introduced and several clinical trials have now published preliminary data in several autoimmune diseases such as psoriasis^47^ and rheumatoid arthritis^45^.

The role of CD6 and ALCAM in ocular inflammatory disease has not received much attention until now, although expression of ALCAM has been described for retinal vascular endothelial cells^48^. ALCAM expression on the retinal vascular endothelium is probably involved in neovascularization and not directly on the transmigration of inflammatory T cells into the retina^8^. Our finding concerning an association of CD6 polymorphisms with BD may therefore be related to an effect of CD6 variants on T cell activation rather than on the egress of these cells into the retina. We could not find an effect of the rs11230563 CD6 on the mRNA expression by PBMCs, but as mentioned above, it might directly influence CD6 function^39^. Further experiments are needed to unravel the functional role of CD6 variants in BD pathogenesis.

It is worthwhile to point out that the odds ratios were toward different directions between the homozygotes and heterozygotes. The genotype data may be due to the dominant behavior of the A allele of rs2929 and T allele of rs11230563. In view of the relatively low frequency of the AA genotype (rs2929) and the TT genotype (rs11230563) both in patients and controls, we did not find a protective effect of the AA genotype of rs2929 (Pc > 0.05, OR = 0.78) or a positive predisposing effect of the TT genotype of rs11230563 (Pc > 0.05, OR = 1.74).

The present study has some limitations that should be noted. Firstly, though we tried to match the controls for gender, 82.9 percent of our BD cases were male, while 54.9 percent of controls were male. Analysis of our data according to gender however showed that the association between rs11230563 and rs2929 and BD was seen in both the female and male population. Secondly, our study identified rs2929 of CD11c and rs11230563 of CD6 as potential risk factors in the susceptibility for BD in a Chinese Han population, but the exact mechanism whereby these variants affect the disease pathogenesis were not clarified and deserves further study. Functional experiments showed that the CD11c risk variant influenced CD11c expression and the production of several pro-inflammatory cytokines. We only examined the effect of rs2929 in healthy genotyped controls because the BD patient population is extremely heterogeneous due to a variable inflammatory course and due to the immunosuppressive drug treatment used. Thirdly, we only chose previously reported loci in the family of adhesion molecules, which were known to be associated with autoimmune diseases and it cannot be ruled out that other SNPs in cell adhesion molecules can be associated with BD. A detailed sequence analysis of the identified risk genes should be performed to investigate whether rare variants of these factors might also be involved, thus strengthening the functional role of these factors in BD development. Last but not the least, since our BD patients were recruited from an ophthalmology department, further confirmations should be done by investigating BD patients originating from other medical fields such as the rheumatology or dermatology departments.

In summary, our study showed that CD6 rs11230563 and CD11c rs2929 polymorphisms are associated with susceptibility to BD in a Chinese Han population. Further studies are needed to reveal the mechanisms whereby CD6 and CD11c expression and function are regulated in BD and whether this knowledge can be used to develop novel therapeutic approaches.

## Methods

### Participants

From April 2008 to October 2015, 1149 BD patients, visiting the First Affiliated Hospital of Chongqing Medical University (Chongqing, China), were selected as the study population. BD patients were strictly diagnosed based upon the criteria of the International Study Group for BD^49^. A group of 2107 healthy individuals, who matched ethnically (Han Chinese) and geographically with the patients, served as the control group. A two-stage case-control association study was carried out. The first-stage study cohort consisted of 391 BD patients and 603 normal controls. In the second stage, a different set of 758 BD patients and 1504 normal controls were enrolled. The study received the approval of the First Affiliated Hospital of Chongqing Medical University Ethics Research Committee and all the investigated subjects provided informed consent before collection of blood. The tenets of the Declaration of Helsinki (2013) were adhered to during all procedures of this study. All methods were carried out in accordance with the approved guidelines.

### Single nucleotide polymorphisms selection

Single nucleotide polymorphism (SNP) selection was based on previous studies on the association between cell adhesion molecules and inflammatory and autoimmune disease^9^,^10^,^11^,^12^,^13^,^14^,^15^,^16^,^17^,^18^,^19^,^20^,^21^,^22^,^23^,^24^,^25^,^26^,^27^,^28^,^29^,^30^,^31^,^32^,^33^,^34^,^35^. Linkage disequilibrium (LD) and Minor Allele Frequency (MAF) were analyzed by Haploview 4.2 software. We eliminated several SNPs which were not polymorphic in the Chinese population and finally selected 43 SNPs (the MAF at each locus was required to be >0.05 in Han Chinese in Beijing, with an r^2^-value of LD <0.8 between adjacent markers) in 26 molecules. The SNPs tested in this study included 4 SNPs (rs281432, rs5498, rs3093030, rs281437) of ICAM1^9^,^10^,^12^, 1 SNP (rs2278442) of ICAM3^11^, 1 SNP (rs2228615) of ICAM5^12^, 3 SNPs (rs3738919, rs3768777, rs3911238) of ITGAV^13^,^14^,^15^, 1 SNP (rs3809865) of ITGB3^16^, 1 SNP (rs886774) of LAMB1^17^;1 SNP (rs6437585) of ALCAM^18^, 5 SNPs (rs1777241, rs1078621, rs7203337, rs10431923, rs1728785) of CDH1^19^,^20^, 1 SNP (rs1417210) of CDH23^21^, 1 SNP (rs6967330) of CDHR3^22^, 1 SNP (rs11150610) of ITGAM^23^, 1 SNP (rs10800469) of E-selectin^24^, 1 SNP (rs2205849) of L-selectin^25^, 1 SNP (rs3917657) of P-selection^26^, 2 SNPs (rs12288280, rs11230563) of CD6^27^,^28^, 1 SNP (rs11574944) of CD11a^29^, 2 SNPs (rs2230429, rs2929) of CD11c^29^, 1 SNP (rs235326) of CD18^29^, 1 SNP (rs1980422) of CD28^30^, 2 SNPs (rs736374, rs10768122) of CD44^31^, 1 SNP (rs4656958) of CD48^28^, 2 SNPs (rs2300747, rs11586238) of CD58^11^,^30^, 4 SNPs (rs4688013, rs2222631, rs59374417, rs4330287) of CD80^31^,^32^,^33^, 1 SNP (rs4308217) of CD86^11^, 1 SNP (rs2891) of CD103^34^ and 2 SNPs (rs763361, rs727088) of CD226^28^,^35^.

### DNA extraction and genotyping

Genomic DNA was extracted from venous blood samples of BD patients and healthy controls using the QIAamp DNA Blood Mini Kit (Qiagen, Valencia, California, USA). All SNPs were assayed using a matrix-assisted laser desorption/ionization time of flight mass spectrometry platform (Sequenom, San Diego, CA) in the first stage, following the manufacturer’s instruction. Samples were genotyped by polymerase chain reaction-restriction fragment length polymorphism (PCR-RFLP) for rs11230563 in the second stage. Digestion products were separated on a 4% agarose gel and stained with GoldView TM (SBS Genetech, Beijing, China). Rs2929 (TagMan assay ID: C_9607211_10) genotypes were analyzed using the TaqMan^®^ SNP Genotyping Assay (Applied Biosystems, Foster City, CA, USA) on the Applied Biosystems 7500 Real-Time PCR system in the second stage. The analysis was conducted using TaqMan^®^ Genotyper Software. Five percent of study samples in a random fashion were sequenced directly to assure the validity of the SNP genotyping method used. The success rate of all SNPs genotyping ranged from 98.0% to 100%.

### Cell isolation and culture

Peripheral blood mononuclear cells (PBMCs) were isolated from heparinized blood samples of healthy controls by Ficoll-Hypaque density-gradient centrifugation. Isolated PBMCs (1 × 10^6^ cells per well) were seeded in 24-well plates and cultured in RPMI medium 1640 supplemented with 10% fetal calf serum (FCS, Greiner, Wemmel, Belgium), 100 U/ml penicillin, 100ug/ml streptomycin. In order to detect the production of IL-1beta, IL-6, IL-8, IL-10, TNF-α and MCP-1, PBMCs were cultured with 100 ng/ml lipopolysaccharide (LPS, L2880, Sigma, Missouri, USA) for 24 hours.

### Real-time polymerase chain reaction

Total RNA was extracted from unstimulated PBMCs and LPS stimulated PBMCs with TRIzol (Invitrogen, Carlsbad, CA) followed by reverse transcription using a transcriptase kit. The sequences of the sense and antisense primers were as follows: CD6: 5′-TGCACAATCTGTCCACTCCC-3′ and 5′-AACGATGGAGGGGATGAGGA-3′; CD11c: 5′-CCGACCATATCTGCCAGGAC-3′ and 5′-CCCGTCATTCCACACCATCA-3′. β-actin was selected as the internal reference gene and its expression was measured by the following primers: forward 5′-GGATGCAGAAGGAGATCACTG-3′ and reverse 5′-CGATCCACACGGAGTACTT-3′. The assays were performed on a 7500 real-time instrument (ABI). Relative expression levels were calculated by the 2−ΔΔCt method.

### Enzyme linked immunosorbent Assay (ELISA)

The concentration of IL-1beta, IL-6, IL-8, IL-10, TNF-α and MCP-1 in PBMC culture supernatants was detected by the human Duoset ELISA development kit (R&D Systems, Minneapolis, Minnesota, USA) based upon the manufacturer’s protocols.

### Statistical analysis

As to SNP analysis, Hardy-Weinberg equilibrium (HWE) was tested by the Chi-square test. Genotype frequencies were calculated by direct counting. Allele and genotype frequencies were compared between patients and controls by the chi-square test using SPSS version 19.0. P values were evaluated with the χ2 test or Fisher’s exact test. Genotype testing was performed by comparing one genotype versus the other two pooled together. P values were corrected (Pc) for multiple comparisons with the Bonferroni correction by multiplying with the number of analyses performed according to the methods of Jiang *et al.*^50^ and Fang *et al.*^51^, and Pc <0.05 was considered statistically significant. Risks were assessed by odds ratios (ORs) with 95% confidence intervals (CIs). To investigate whether associations could be elaborated by either dominant or recessive models, the data of rs2929 and rs11230563 genotype frequencies were analyzed by Univariate logistic regression and Multivariate logistic regression. The non-parametric Mann-Whitney test was used to compare CD6, CD11c expression and cytokine levels among three genotype groups.

## Additional Information

**How to cite this article**: Zheng, M. *et al.* Genetic polymorphisms of cell adhesion molecules in Behcet's disease in a Chinese Han population. *Sci. Rep.* **6**, 24974; doi: 10.1038/srep24974 (2016).

## Supplementary Material

Supplementary Information

## Figures and Tables

**Table 1:** Clinical characteristics, gender and age of BD patients with uveitis.

Clinical features	Total	%
Patients with BD		
Mean age ± SD	34.0 ± 9.1	
Male	952	82.9
Female	197	17.1
Uveitis	1149	100
Oral ulcer	1067	92.9
Genital ulcer	629	54.7
Skin lesion	846	73.6
Arthritis	205	17.8
Pathergy reaction	234	20.4
Controls		
Mean age ± SD	39.6 ± 11.7	
Male	1156	54.9
Female	951	45.1

**Table 2:** Association of two SNPs with Behçet’s Disease.

Gene	SNP	Stage	Genotype/Allele	BD		Controls		P Value	Pc Value	OR (95% CI)
				N	%	N	%			
ITGAX (CD11c)	rs2929	First	AA	15	3.8	23	3.8	0.986	NS	1.01 (0.52–1.95)
			AG	82	21.0	193	32.0	1.453 × 10^−4^	0.019	0.56 (0.42–0.76)
			GG	294	75.2	387	64.2	2.606 × 10^−4^	0.034	1.69 (1.27–2.25)
			G	670	85.7	967	80.2	0.002	NS	1.48 (1.16–1.89)
		Second	AA	12	1.6	40	2.7	0.107	NS	0.59 (0.31–1.13)
			AG	180	23.7	470	31.3	1.974 × 10^−4^	1.184 × 10^−3^	0.69 (0.56–0.84)
			GG	566	74.7	994	66.1	3.135 × 10^−5^	1.881 × 10^−4^	1.51 (1.24–1.84)
			G	1312	86.5	2458	81.7	3.904 × 10^−5^	7.808 × 10^−5^	1.44 (1.21–1.71)
		Combined	AA	27	2.3	63	3.0	0.287	NS	0.78 (0.50–1.23)
			AG	262	22.8	663	31.5	1.618 × 10^−7^	2.087 × 10^−5^	0.64 (0.55–0.76)
			GG	860	74.8	1381	65.5	4.320 × 10^−8^	5.573 × 10^−6^	1.56 (1.33–1.84)
			G	1982	86.2	3425	81.3	3.252 × 10^−7^	1.398 × 10^−5^	1.45 (1.25–1.67)
CD6	rs11230563	First	CC	252	64.5	468	77.6	5.721 × 10^−6^	7.380 × 10^−4^	0.52 (0.39–0.69)
			CT	128	32.7	121	20.1	6.685 × 10^−6^	8.624 × 10^−4^	1.94 (1.45–2.59)
			TT	11	2.8	14	2.3	0.629	NS	1.22 (0.55–2.71)
			C	632	80.8	1057	87.6	3.189 × 10^−5^	1.371 × 10^−3^	0.59 (0.46–0.76)
		Second	CC	501	66.1	1137	75.6	1.811 × 10^−6^	1.087 × 10^−5^	0.63 (0.52–0.76)
			CT	238	31.4	349	23.2	2.714 × 10^−5^	1.628 × 10^−4^	1.52 (1.25–1.84)
			TT	19	2.5	18	1.2	0.020	NS	2.12 (1.11–4.07)
			C	1240	81.8	2623	87.2	1.176 × 10^−6^	2.352 × 10^−5^	0.66 (0.56–0.78)
		combined	CC	753	65.5	1605	76.2	8.501 × 10^−11^	1.097 × 10^−8^	0.60 (0.51–0.70)
			CT	366	31.9	470	22.3	2.532 × 10^−9^	3.266 × 10^−7^	1.63 (1.39–1.91)
			TT	30	2.6	32	1.5	0.029	NS	1.74 (1.05–2.88)
			C	1872	81.5	3680	87.3	1.766 × 10^−10^	7.594 × 10^−9^	0.64 (0.56–0.73)

Pc, Bonferroni corrected p value; NS, not significant; SNP, single nucleotide polymorphism.

**Table 3:** The distribution of two SNPs in patients with BD and healthy controls by gender basis.

SNP	Allele/Genotype	Male		P Value	Pc Value	OR (95% CI)	Female		P Value	Pc Value	OR (95% CI)
		BD (n = 952)	Controls (n = 1156)				BD (n = 197)	Controls (n = 951)			
rs2929	AA	24	36	NS	NS	0.81 (0.48–1.36)	3	27	NS	NS	0.53 (0.16–1.76)
	AG	219	349	2.149 × 10^−4^	1.289 × 10^−3^	0.69 (0.57–0.84)	43	314	0.002	0.012	0.57 (0.39–0.82)
	GG	709	771	1.017 × 10^−4^	6.102 × 10^−4^	1.46 (1.21–1.76)	151	610	0.001	0.006	1.84 (1.29–2.62)
	G	1637	1891	2.517 × 10^−4^	5.034 × 10^−4^	1.37 (1.16–1.61)	345	1534	0.001	0.002	1.69 (1.23–2.33)
rs11230563	CC	624	883	4.133 × 10^−8^	2.480 × 10^−7^	0.59 (0.49–0.71)	129	722	0.002	0.012	0.60 (0.43–0.84)
	CT	305	256	3.144 × 10^−7^	1.886 × 10^−6^	1.66 (1.36–2.01)	61	214	0.011	NS	1.55 (1.10–2.17)
	TT	23	17	NS	NS	1.66 (0.88–3.12)	7	15	NS	NS	2.30 (0.93–5.72)
	C	1553	2022	1.145 × 10^−7^	2.290 × 10^−7^	0.64 (0.54–0.75)	319	1658	0.001	0.002	0.63 (0.47–0.83)

NS, not significant; SNP, single nucleotide polymorphism; BD: Behçet’s Disease.

**Table 4:** Logistic regression analysis of the risk of BD patients with CD11c/rs2929 in additive co-dominant, dominant and recessive models.

Model	Genotype	Control (*N* = 2107)	Case (*N* = 1149)	Univariate logistic regression		Multivariate logistic regression^a^	
				*OR*(95% *CI*)	*P*^b^	*OR*(95% *CI*)	*P*^b^
Additive				0.6892 (0.5973,0.7951)	<0.0001	0.7052 (0.6055,0.8213)	<0.0001
Co-dominant	GG	1381 (65.54%)	860 (74.85%)	Ref.		Ref.	
	AG	663 (31.47%)	262 (22.80%)	0.6346 (0.5373,0.7495)	<0.0001	0.6436 (0.5389,0.7688)	<0.0001
	AA	63 (2.99%)	27 (2.35%)	0.6882 (0.4350,1.0889)	0.1104	0.7455 (0.4566,1.2172)	0.2403
Dominant	GG	1381 (65.54%)	860 (74.85%)	Ref.		Ref.	
	AG+AA	726 (34.46%)	289 (25.15%)	0.6393 (0.5444,0.7507)	<0.0001	0.6521 (0.5493,0.7742)	<0.0001
Recessive	GG+AG	2044 (97.01%)	1122 (97.65%)	Ref.		Ref.	
	AA	63 (2.99%)	27 (2.35%)	0.7808 (0.4945,1.2327)	0.2882	0.8422 (0.5169,1.3721)	0.4903

95% CI 95% confidence interval, OR odds ratio.

^a^The age, sex were adjusted in the multivariate logistic regression model;

^b^The hypothesis test was performed using Wald *χ*^2^ test.

**Table 5:** Logistic regression analysis of the risk of BD patients with CD6/rs11230563 in additive co-dominant, dominant and recessive models.

Model	Genotype	Control (*N* = 2107)	Case (*N* = 1149)	Univariate logistic regression		Multivariate logistic regression^a^	
				*OR*(95%* CI*)	*P*^b^	*OR*(95%* CI*)	*P*^b^
Additive				1.5890 (1.3787,1.8313)	<0.0001	1.6129 (1.3839,1.8797)	<0.0001
Co-dominant	CC	1605 (76.17%)	753 (65.54%)	Ref.		Ref.	
	CT	470 (22.31%)	366 (31.85%)	1.6598 (1.4120,1.9513)	<0.0001	1.6494 (1.3855,1.9635)	<0.0001
	TT	32 (1.52%)	30 (2.61%)	1.9983 (1.2053,3.3130)	0.0073	2.3023 (1.3293,3.9877)	0.0029
Dominant	CC	1605 (76.17%)	753 (65.54%)	Ref.		Ref.	
	CT+TT	502 (23.83%)	396 (34.46%)	1.6814 (1.4362,1.9685)	<0.0001	1.6874 (1.4237,2.0000)	<0.0001
Recessive	CC+CT	2075 (98.48%)	1119 (97.39%)	Ref.		Ref.	
	TT	32 (1.52%)	30 (2.61%)	1.7385 (1.0509,2.8759)	0.0313	2.0056 (1.1607,3.4656)	0.0126

95% CI 95% confidence interval, OR odds ratio.

^a^The age, sex were adjusted in the multivariate logistic regression model;

^b^The hypothesis test was performed using Wald *χ*^2^ test.
