# Supplementary material for: Genetic polymorphisms of cell adhesion molecules in Behcet’s disease in a Chinese Han population
Source: Sci Rep. 2016 Apr 25;6:24974. doi: 10.1038/srep24974 (PMC4842956; doi:10.1038/srep24974)
Supplement: Supplementary Information [file srep24974-s1.doc]

**Genetic polymorphisms of cell adhesion molecules in** [**Behcet's disease**](http://www.ncbi.nlm.nih.gov/pubmed/26269006) [**in**](http://www.ncbi.nlm.nih.gov/pubmed/26136352) **a Chinese Han population**

Minming Zheng^1*^; Lijun Zhang^1*^; Hongsong Yu^1*^; Jiayue Hu^1^; Qingfeng Cao^1^;Guo Huang^1^; Yang Huang^1^; Gangxiang Yuan^1^; Aize Kijlstra^2^; Peizeng Yang^1^

^1^ The First Affiliated Hospital of Chongqing Medical University, Chongqing Key Laboratory of Ophthalmology and Chongqing Eye Institute, Chongqing, P. R. China

^2^ University Eye Clinic Maastricht, Maastricht, The Netherlands

Correspondence to: Professor Peizeng Yang, MD, Ph.D.,

Department of Ophthalmology

The First Affiliated Hospital of Chongqing Medical University

Chongqing Key Laboratory of Ophthalmology and Chongqing Eye Institute

1 You Yi Road, Yu Zhong District

Chongqing, 400016 China

Phone: +8623 89012851;

FAX: +8623 89012851;

Email: [peizengycmu@126.com](mailto:peizengycmu@126.com)

* These authors contributed equally to this work.

| **Table S1 Polymorphisms of other CAM genes in BD** | | | | | | | | | |
| --- | --- | --- | --- | --- | --- | --- | --- | --- | --- |
| **Gene** | **SNP** | **Genotype/Allele** | **BD** | | **CN** | | **P for BD** | **Pc** | **OR(95% CI)** |
|  |  |  | **N** | **%** | **N** | **%** |  |  |  |
| CD6 | rs12288280 | GG | 319 | 82.4 | 511 | 84.7 | 0.334 | NS | 0.85(0.60-1.19) |
|  |  | GT | 66 | 17.1 | 87 | 14.4 | 0.365 | NS | 1.22(0.86-1.73) |
|  |  | TT | 2 | 0.5 | 5 | 0.8 | 0.567 | NS | 0.62(0.12-3.22) |
|  |  | G | 704 | 91.0 | 1109 | 92.0 | 0.434 | NS | 0.88(0.64-1.21) |
| CD11a | rs11574944 | TT | 3 | 0.8 | 14 | 2.3 | 0.066 | NS | 0.33(0.09-1.15) |
|  |  | CT | 81 | 20.8 | 139 | 23.1 | 0.409 | NS | 0.88(0.64-1.20) |
|  |  | CC | 305 | 78.4 | 450 | 74.6 | 0.173 | NS | 1.24(0.91-1.67) |
|  |  | C | 691 | 88.8 | 1039 | 86.2 | 0.083 | NS | 1.28(0.97-1.68) |
| ITGAX(CD11c) | rs2230429 | CC | 350 | 89.5 | 550 | 91.2 | 0.372 | NS | 0.82(0.54-1.26) |
|  |  | GC | 41 | 10.5 | 51 | 8.5 | 0.281 | NS | 1.27(0.82-1.95) |
|  |  | GG | 0 | 0.0 | 2 | 0.3 | - | - | - |
|  |  | C | 741 | 94.8 | 1151 | 95.4 | 0.488 | NS | 0.86(0.57-1.31) |
| CD18 | rs235326 | AA | 14 | 3.6 | 29 | 4.8 | 0.374 | NS | 0.75(0.39-1.43) |
|  |  | AG | 124 | 32.1 | 195 | 32.3 | 0.944 | NS | 0.99(0.75-1.30) |
|  |  | GG | 248 | 64.2 | 379 | 62.9 | 0.657 | NS | 1.06(0.81-1.39) |
|  |  | G | 620 | 80.3 | 953 | 79.0 | 0.488 | NS | 1.08(0.87-1.36) |
| CD28 | rs1980422 | CC | 4 | 1.0 | 8 | 1.3 | 0.686 | NS | 0.78(0.23-2.61) |
|  |  | CT | 64 | 16.7 | 111 | 18.5 | 0.470 | NS | 0.88(0.63-1.24) |
|  |  | TT | 316 | 82.3 | 482 | 80.2 | 0.414 | NS | 1.15(0.83-1.60) |
|  |  | C | 72 | 9.4 | 127 | 10.6 | 0.392 | NS | 0.88(0.65-1.19) |
| CD44 | rs10768122 | AA | 107 | 27.4 | 169 | 28.0 | 0.820 | NS | 0.97(0.73-1.29) |
|  |  | AG | 206 | 52.7 | 294 | 48.8 | 0.226 | NS | 1.17(0.91-1.51) |
|  |  | GG | 78 | 19.9 | 140 | 23.2 | 0.224 | NS | 0.82(0.60-1.13) |
|  |  | A | 420 | 53.7 | 632 | 52.4 | 0.569 | NS | 1.05(0.88-1.26) |
| CD44 | rs736374 | AA | 79 | 20.3 | 140 | 23.2 | 0.272 | NS | 0.84(0.62-1.15) |
|  |  | AG | 204 | 52.3 | 294 | 48.8 | 0.274 | NS | 1.15(0.89-1.49) |
|  |  | GG | 107 | 27.4 | 169 | 28.0 | 0.839 | NS | 0.97(0.73-1.29) |
|  |  | G | 418 | 53.6 | 632 | 52.4 | 0.605 | NS | 1.05(0.88-1.26) |
| CD48 | rs4656958 | AA | 33 | 8.4 | 39 | 6.5 | 0.241 | NS | 1.33(0.82-2.16) |
|  |  | GA | 144 | 36.8 | 231 | 38.3 | 0.638 | NS | 0.94(0.72-1.22) |
|  |  | GG | 214 | 54.7 | 333 | 55.2 | 0.879 | NS | 0.98(0.76-1.27) |
|  |  | G | 572 | 73.1 | 897 | 74.4 | 0.541 | NS | 0.94(0.77-1.15) |
| CD58 | rs2300747 | GG | 135 | 35.1 | 214 | 35.5 | 0.892 | NS | 0.98(0.75-1.28) |
|  |  | GA | 188 | 48.8 | 280 | 46.4 | 0.462 | NS | 1.10(0.85-1.42) |
|  |  | AA | 62 | 16.1 | 109 | 18.1 | 0.424 | NS | 0.87(0.62-1.23) |
|  |  | G | 458 | 59.5 | 708 | 58.7 | 0.733 | NS | 1.03(0.86-1.24) |
| CD2-CD58 | rs11586238 | CC | 352 | 90.0 | 561 | 93.2 | 0.073 | NS | 0.66(0.42-1.04) |
|  |  | CG | 37 | 9.5 | 41 | 6.8 | 0.129 | NS | 1.43(0.90-2.27) |
|  |  | GG | 2 | 0.5 | 0 | 0.0 | - | - | - |
|  |  | C | 741 | 94.8 | 1163 | 96.6 | 0.044 | NS | 0.64(0.41-0.99) |
| C3orfl/CD80 | rs4688013 | AA | 39 | 10.0 | 58 | 9.7 | 0.867 | NS | 1.04(0.68-1.59) |
|  |  | GA | 151 | 38.6 | 255 | 42.4 | 0.233 | NS | 0.85(0.66-1.11) |
|  |  | GG | 201 | 51.4 | 288 | 47.9 | 0.283 | NS | 1.15(0.89-1.48) |
|  |  | A | 229 | 29.3 | 371 | 30.9 | 0.454 | NS | 0.93(0.76-1.13) |
| CD80 | rs2222631 | AA | 112 | 29.1 | 157 | 26.4 | 0.354 | NS | 1.15(0.86-1.52) |
|  |  | AG | 192 | 49.9 | 296 | 49.7 | 0.970 | NS | 1.01(0.78-1.30) |
|  |  | GG | 81 | 21.0 | 142 | 23.9 | 0.303 | NS | 0.85(0.62-1.16) |
|  |  | A | 416 | 54.0 | 610 | 51.3 | 0.231 | NS | 1.12(0.93-1.34) |
| CD80 | rs4330287 | AA | 29 | 7.4 | 43 | 7.1 | 0.865 | NS | 1.05(0.64-1.71) |
|  |  | CA | 141 | 36.2 | 248 | 41.1 | 0.117 | NS | 0.81(0.62-1.050 |
|  |  | CC | 220 | 56.4 | 312 | 51.7 | 0.150 | NS | 1.21(0.93-1.560 |
|  |  | C | 581 | 74.5 | 872 | 72.3 | 0.618 | NS | 0.95(0.79-1.15) |
| CD80 | rs59374417 | AA | 219 | 56.2 | 312 | 51.7 | 0.173 | NS | 1.20(0.93-1.54) |
|  |  | CA | 141 | 36.2 | 252 | 41.8 | 0.076 | NS | 0.79(0.61-1.03) |
|  |  | CC | 30 | 7.7 | 39 | 6.5 | 0.459 | NS | 1.21(0.74-1.98) |
|  |  | A | 579 | 74.2 | 876 | 72.6 | 0.433 | NS | 1.09(0.89-1.33) |
| CD86 | rs4308217 | CC | 273 | 70.9 | 445 | 73.8 | 0.320 | NS | 0.87(0.65-1.15) |
|  |  | CA | 101 | 26.2 | 148 | 24.5 | 0.551 | NS | 1.09(0.82-1.47) |
|  |  | AA | 11 | 2.9 | 10 | 1.7 | 0.203 | NS | 1.74(0.73-4.15) |
|  |  | C | 647 | 84.0 | 1038 | 86.1 | 0.211 | NS | 0.85(0.66-1.10) |
| CD103 | rs2891 | TT | 60 | 15.5 | 69 | 11.4 | 0.064 | NS | 1.42(0.98-2.06) |
|  |  | TC | 165 | 42.6 | 292 | 48.4 | 0.075 | NS | 0.79(0.61-1.02) |
|  |  | CC | 162 | 41.9 | 242 | 40.1 | 0.589 | NS | 1.07(0.83-1.39) |
|  |  | C | 489 | 63.2 | 776 | 64.3 | 0.598 | NS | 0.95(0.79-1.15) |
| CD226 | rs727088 | AA | 214 | 54.7 | 322 | 53.4 | 0.681 | NS | 1.06(0.82-1.36) |
|  |  | AG | 145 | 37.1 | 247 | 41.0 | 0.222 | NS | 0.85(0.65-1.10) |
|  |  | GG | 32 | 8.2 | 34 | 5.6 | 0.115 | NS | 1.49(0.90-2.46) |
|  |  | A | 573 | 73.3 | 891 | 73.9 | 0.764 | NS | 0.97(0.79-1.19) |
| CD226 | rs763361 | TT | 41 | 10.6 | 59 | 9.8 | 0.670 | NS | 1.10(0.72-1.67) |
|  |  | TC | 184 | 47.7 | 282 | 46.8 | 0.782 | NS | 1.04(0.80-1.34) |
|  |  | CC | 161 | 41.7 | 262 | 43.4 | 0.590 | NS | 0.93(0.72-1.21) |
|  |  | C | 506 | 65.5 | 806 | 66.8 | 0.554 | NS | 0.94(0.78-1.14) |
| L-selectin(CD62L) | rs2205849 | CC | 35 | 9.0 | 59 | 9.8 | 0.661 | NS | 0.91(0.58-1.41) |
|  |  | CT | 188 | 48.1 | 249 | 41.3 | 0.035 | NS | 1.31(1.02-1.70) |
|  |  | TT | 168 | 43.0 | 295 | 48.9 | 0.066 | NS | 0.79(0.61-1.02) |
|  |  | C | 258 | 33.0 | 367 | 30.4 | 0.230 | NS | 1.13(0.93-1.37) |
| E-selectin(CD62E) | rs10800469 | GG | 147 | 38.1 | 219 | 36.3 | 0.575 | NS | 1.08(0.83-1.40) |
|  |  | AG | 182 | 47.2 | 294 | 48.8 | 0.622 | NS | 0.94(0.73-1.21) |
|  |  | AA | 57 | 14.8 | 90 | 14.9 | 0.945 | NS | 0.99(0.69-1.42) |
|  |  | G | 476 | 61.7 | 732 | 60.7 | 0.669 | NS | 1.04(0.87-1.25) |
| P-selectin(CD62P) | rs3917657 | GG | 240 | 62.7 | 390 | 64.7 | 0.521 | NS | 0.92(0.70-1.20) |
|  |  | GA | 121 | 31.6 | 181 | 30.0 | 0.601 | NS | 1.08(0.82-1.42) |
|  |  | AA | 22 | 5.7 | 32 | 5.3 | 0.769 | NS | 1.09(0.62-1.90) |
|  |  | G | 601 | 78.5 | 961 | 79.7 | 0.513 | NS | 0.93(0.74-1.16) |
| ICAM-1 | rs281432 | GG | 37 | 9.6 | 51 | 8.7 | 0.613 | NS | 1.12(0.72-1.75) |
|  |  | GC | 164 | 42.6 | 260 | 44.1 | 0.634 | NS | 0.94(0.73-1.22) |
|  |  | CC | 184 | 47.8 | 278 | 47.2 | 0.856 | NS | 1.02(0.78-1.32) |
|  |  | C | 532 | 69.1 | 816 | 69.3 | 0.933 | NS | 0.99(0.82-1.21) |
| ICAM-1 | rs5498 | AA | 164 | 42.5 | 305 | 50.6 | 0.013 | NS | 0.72(0.56-0.93) |
|  |  | AG | 187 | 48.4 | 248 | 41.1 | 0.024 | NS | 1.35(1.04-1.74) |
|  |  | GG | 35 | 9.1 | 50 | 8.3 | 0.671 | NS | 1.10(0.70-1.73) |
|  |  | A | 515 | 66.7 | 858 | 71.1 | 0.037 | NS | 0.81(0.67-0.99） |
| ICAM-3 | rs2278442 | AA | 229 | 58.6 | 366 | 60.8 | 0.484 | NS | 0.91(0.70-1.18) |
|  |  | GA | 136 | 34.8 | 196 | 32.6 | 0.468 | NS | 1.11(0.84-1.45) |
|  |  | GG | 26 | 6.6 | 40 | 6.6 | 0.997 | NS | 1.00(0.60-1.67) |
|  |  | A | 594 | 76.0 | 928 | 77.1 | 0.565 | NS | 0.94(0.76-1.16) |
| ICAM1-ICAM4 | rs228615 | TT | 123 | 31.5 | 179 | 29.7 | 0.553 | NS | 1.09(0.83-1.43) |
|  |  | TA | 178 | 45.5 | 300 | 49.8 | 0.193 | NS | 0.84(0.65-1.09) |
|  |  | AA | 90 | 23.0 | 124 | 20.6 | 0.358 | NS | 1.16(0.85-1.57) |
|  |  | T | 424 | 54.2 | 658 | 54.6 | 0.882 | NS | 0.99(0.82-1.18) |
| ICAM1-ICAM4 | rs281437 | CC | 317 | 80.9 | 474 | 78.6 | 0.388 | NS | 1.15(0.84-1.58) |
|  |  | CT | 68 | 17.3 | 121 | 20.1 | 0.285 | NS | 0.84(0.60-1.16) |
|  |  | TT | 7 | 1.8 | 8 | 1.3 | 0.562 | NS | 1.35(0.49-3.76) |
|  |  | C | 702 | 89.5 | 1069 | 88.6 | 0.530 | NS | 1.10(0.82-1.47) |
| ICAM1-ICAM4 | rs3093030 | TT | 33 | 8.5 | 32 | 5.3 | 0.052 | NS | 1.64(0.99-2.72) |
|  |  | TC | 148 | 38.0 | 229 | 38.2 | 0.954 | NS | 0.99(0.76-1.29) |
|  |  | CC | 208 | 53.5 | 338 | 56.4 | 0.361 | NS | 0.89(0.69-1.15) |
|  |  | C | 564 | 72.5 | 905 | 75.5 | 0.129 | NS | 0.85(0.70-1.05) |
| ITGAM(CD11b) | rs11150610 | AA | 204 | 52.3 | 304 | 50.6 | 0.596 | NS | 1.07(0.83-1.38) |
|  |  | CA | 152 | 39.0 | 238 | 39.6 | 0.844 | NS | 0.97(0.75-1.27) |
|  |  | CC | 34 | 8.7 | 59 | 9.8 | 0.562 | NS | 0.88(0.56-1.37) |
|  |  | A | 560 | 71.8 | 846 | 70.4 | 0.499 | NS | 1.07(0.88-1.31) |
| ITGAV | rs3738919 | CC | 330 | 84.4 | 519 | 86.1 | 0.466 | NS | 0.88(0.61-1.25) |
|  |  | CA | 61 | 15.6 | 82 | 13.6 | 0.380 | NS | 1.17(0.82-1.68) |
|  |  | AA | 0 | 0.0 | 2 | 0.3 | - | - |  |
|  |  | C | 721 | 92.2 | 1120 | 92.9 | 0.577 | NS | 0.91(0.65-1.28) |
| ITGAV | rs3768777 | AA | 26 | 6.6 | 24 | 4.0 | 0.061 | NS | 1.72(0.97-3.03) |
|  |  | GA | 128 | 32.7 | 190 | 31.6 | 0.698 | NS | 1.06(0.80-1.39) |
|  |  | GG | 237 | 60.6 | 388 | 64.5 | 0.221 | NS | 0.85(0.65-1.10) |
|  |  | G | 602 | 77.0 | 966 | 80.2 | 0.083 | NS | 0.82(0.66-1.03) |
| ITGAV | rs3911238 | CC | 196 | 50.3 | 316 | 53.6 | 0.311 | NS | 0.88(0.68-1.13) |
|  |  | CG | 154 | 39.5 | 227 | 38.5 | 0.750 | NS | 1.04(0.80-1.36) |
|  |  | GG | 40 | 10.3 | 47 | 8.0 | 0.217 | NS | 1.32(0.85-2.06) |
|  |  | C | 546 | 70.0 | 859 | 72.8 | 0.179 | NS | 0.87(0.71-1.07) |
| ITGB3 | rs3809865 | AA | 251 | 65.0 | 385 | 63.8 | 0.706 | NS | 1.05(0.81-1.38) |
|  |  | AT | 122 | 31.6 | 191 | 31.7 | 0.982 | NS | 1.00(0.76-1.31) |
|  |  | TT | 13 | 3.4 | 27 | 4.5 | 0.387 | NS | 0.74(0.38-1.46) |
|  |  | A | 624 | 80.8 | 961 | 79.7 | 0.534 | NS | 1.08(0.86-1.35) |
| LAMB1 | rs886774 | AA | 250 | 63.9 | 360 | 59.7 | 0.180 | NS | 1.20(0.92-1.56) |
|  |  | AG | 124 | 31.7 | 215 | 35.7 | 0.200 | NS | 0.84(0.64-1.10) |
|  |  | GG | 17 | 4.3 | 28 | 4.6 | 0.827 | NS | 0.93(0.50-1.73) |
|  |  | A | 624 | 79.8 | 935 | 77.5 | 0.230 | NS | 1.15(0.92-1.43) |
| ALCAM | rs6437585 | TT | 6 | 1.6 | 11 | 1.8 | 0.746 | NS | 0.85(0.31-2.31) |
|  |  | TC | 93 | 24.0 | 136 | 22.6 | 0.591 | NS | 1.09(0.80-1.47) |
|  |  | CC | 288 | 74.4 | 456 | 75.6 | 0.669 | NS | 0.94(0.70-1.26) |
|  |  | C | 669 | 86.4 | 1048 | 86.9 | 0.766 | NS | 0.96(0.74-1.25) |
| CDH1 | rs1728785 | AA | 10 | 2.6 | 27 | 4.5 | 0.118 | NS | 0.56(0.27-1.17) |
|  |  | AC | 114 | 29.2 | 169 | 28.0 | 0.700 | NS | 1.06(0.80-1.40) |
|  |  | CC | 267 | 68.3 | 407 | 67.5 | 0.794 | NS | 1.04(0.79-1.36) |
|  |  | C | 648 | 82.9 | 983 | 81.5 | 0.442 | NS | 1.10(0.87-1.39) |
| CDH1 | rs17772411 | CC | 27 | 6.9 | 41 | 6.8 | 0.948 | NS | 1.02(0.62-1.68) |
|  |  | CT | 124 | 31.7 | 232 | 38.5 | 0.030 | NS | 0.74(0.57-0.97) |
|  |  | TT | 240 | 61.4 | 330 | 54.7 | 0.038 | NS | 1.32(1.02-1.70) |
|  |  | C | 178 | 22.8 | 314 | 26.0 | 0.098 | NS | 0.84(0.68-1.03) |
| CDH1 | rs7203337 | CC | 114 | 29.7 | 187 | 31.6 | 0.530 | NS | 0.91(0.69-1.21) |
|  |  | CG | 211 | 54.9 | 302 | 51.0 | 0.229 | NS | 1.17(0.91-1.52) |
|  |  | GG | 59 | 15.4 | 103 | 17.4 | 0.404 | NS | 0.86(0.61-1.22) |
|  |  | C | 439 | 57.2 | 676 | 57.1 | 0.977 | NS | 1.00(0.84-1.21) |
| CDH1 | rs10431923 | GG | 77 | 19.7 | 124 | 20.6 | 0.744 | NS | 0.95(0.69-1.30) |
|  |  | GT | 207 | 53.1 | 302 | 50.2 | 0.370 | NS | 1.12(0.87-1.45) |
|  |  | TT | 106 | 27.2 | 176 | 29.2 | 0.483 | NS | 0.90(0.68-1.20) |
|  |  | T | 419 | 53.7 | 654 | 54.3 | 0.793 | NS | 0.98(0.82-1.17) |
| CDH1 | rs1078621 | AA | 107 | 27.4 | 144 | 23.9 | 0.208 | NS | 1.21(0.90-1.61) |
|  |  | AG | 197 | 50.5 | 302 | 50.1 | 0.895 | NS | 1.02(0.79-1.31) |
|  |  | GG | 86 | 22.1 | 157 | 26.0 | 0.154 | NS | 0.80(0.60-1.09) |
|  |  | A | 411 | 52.7 | 590 | 48.9 | 0.101 | NS | 1.16(0.97-1.39) |
| CDH23 | rs1417210 | AA | 214 | 55.3 | 300 | 50.4 | 0.135 | NS | 1.22(0.94-1.57) |
|  |  | AG | 127 | 32.8 | 236 | 39.7 | 0.030 | NS | 0.74(0.57-0.97) |
|  |  | GG | 46 | 11.9 | 59 | 9.9 | 0.329 | NS | 1.23(0.81-1.84) |
|  |  | A | 555 | 71.7 | 836 | 70.3 | 0.489 | NS | 1.07(0.88-1.31) |
| CDHR3 | rs6967330 | AA | 5 | 1.3 | 5 | 0.8 | 0.477 | NS | 1.57(0.45-5.44) |
|  |  | AG | 55 | 14.2 | 66 | 10.9 | 0.126 | NS | 1.35(0.92-1.98) |
|  |  | GG | 327 | 84.5 | 532 | 88.2 | 0.091 | NS | 0.73(0.50-1.05) |
|  |  | A | 65 | 8.4 | 76 | 6.3 | 0.077 | NS | 1.36(0.97-1.92) |
| Pc, Bonferroni corrected p value; NS, not significant; SNP, single nucleotide polymorphism | | | | | | | | | |

| **Table S2 Main effects of rs2929/CD11c on clinical feature risk of BD** | | | | | | | | |
| --- | --- | --- | --- | --- | --- | --- | --- | --- |
| **Clinical features** | **Genotype** | **BD with** | | **BD without** | | **P value** | **Pc value** | **OR (95 % CI)** |
|  |  | **N** | **%** | **N** | **%** |  |  |  |
| Genital ulcer |  | 629 |  | 520 |  |  |  |  |
|  | AA | 18 | 2.9 | 9 | 1.7 | 0.208 | NS | 1.67(0.75-3.76) |
|  | AG | 127 | 20.2 | 135 | 26.0 | 0.020 | NS | 0.72(0.55-0.95) |
|  | GG | 484 | 76.9 | 376 | 72.3 | 0.071 | NS | 1.28(0.98-1.67) |
|  | G allele | 1095 | 87.0 | 887 | 85.3 | 0.224 | NS | 1.16(0.91-1.47) |
| Skin lesions |  | 864 |  | 285 |  |  |  |  |
|  | AA | 21 | 2.4 | 6 | 2.1 | 0.753 | NS | 1.16(0.46-2.90) |
|  | AG | 192 | 22.2 | 70 | 24.6 | 0.552 | NS | 1.10(0.81-1.49) |
|  | GG | 651 | 75.4 | 209 | 73.3 | 0.497 | NS | 1.11(0.82-1.51) |
|  | G allele | 1494 | 86.5 | 488 | 85.6 | 0.612 | NS | 1.07(0.82-1.41) |
| Arthritis |  | 205 |  | 944 |  |  |  |  |
|  | AA | 7 | 3.4 | 20 | 2.1 | 0.267 | NS | 1.63(0.68-3.92) |
|  | AG | 49 | 23.9 | 213 | 22.6 | 0.679 | NS | 1.08(0.76-1.54) |
|  | GG | 149 | 72.7 | 711 | 75.3 | 0.431 | NS | 0.87(0.62-1.23) |
|  | G allele | 347 | 84.6 | 1635 | 86.6 | 0.295 | NS | 0.85(0.63-1.15) |
| Positive pathergy reaction |  | 234 |  | 915 |  |  |  |  |
|  | AA | 5 | 2.1 | 22 | 2.4 | 0.809 | NS | 0.89(0.33-2.37) |
|  | AG | 60 | 25.6 | 202 | 22.1 | 0.246 | NS | 1.22(0.87-1.70) |
|  | GG | 169 | 72.2 | 691 | 75.5 | 0.300 | NS | 0.84(0.61-1.17) |
|  | G allele | 398 | 85.0 | 1584 | 86.6 | 0.396 | NS | 0.88(0.66-1.18) |
| Hypopyon |  | 260 |  | 889 |  |  |  |  |
|  | AA | 4 | 1.5 | 23 | 2.6 | 0.330 | NS | 0.59(0.20-1.72) |
|  | AG | 60 | 23.1 | 202 | 22.7 | 0.905 | NS | 1.02(0.74-1.42) |
|  | GG | 196 | 75.4 | 664 | 74.7 | 0.821 | NS | 1.04(0.75-1.43) |
|  | G allele | 452 | 86.9 | 1530 | 86.1 | 0.612 | NS | 1.08(0.81-1.44) |
| NS: not significant; 95 % CI: 95 % confidence interval | | | | | | | | |

| **Table S3 Main effects of rs11230563/CD11c on clinical feature risk of BD** | | | | | | | | |
| --- | --- | --- | --- | --- | --- | --- | --- | --- |
| **Clinical features** | **Genotype** | **BD with** | | **BD without** | | **P value** | **Pc value** | **OR (95 % CI)** |
|  |  | **N** | **%** | **N** | **%** |  |  |  |
| Genital ulcer |  | 629 |  | 520 |  |  |  |  |
|  | CC | 413 | 65.6 | 340 | 65.4 | 0.922 | NS | 1.01(0.79-1.29) |
|  | CT | 201 | 32.0 | 165 | 31.7 | 0.935 | NS | 1.01(0.79-1.30) |
|  | TT | 15 | 2.4 | 15 | 2.9 | 0.597 | NS | 0.82(0.40-1.70) |
|  | C allele | 1027 | 81.6 | 845 | 81.3 | 0.812 | NS | 1.03(0.83-1.27) |
| Skin lesions |  | 864 |  | 285 |  |  |  |  |
|  | CC | 557 | 64.5 | 196 | 68.8 | 0.185 | NS | 0.82(0.62-1.10) |
|  | CT | 282 | 32.6 | 84 | 29.5 | 0.320 | NS | 1.16(0.87-1.55) |
|  | TT | 25 | 2.9 | 5 | 1.7 | 0.296 | NS | 1.67(0.63-4.40) |
|  | C allele | 1396 | 80.8 | 476 | 83.5 | 0.147 | NS | 0.83(0.65-1.07) |
| Arthritis |  | 205 |  | 944 |  |  |  |  |
|  | CC | 137 | 66.8 | 616 | 65.3 | 0.667 | NS | 1.07(0.78-1.48) |
|  | CT | 60 | 29.3 | 306 | 32.4 | 0.381 | NS | 0.86(0.62-1.20) |
|  | TT | 8 | 3.9 | 22 | 2.3 | 0.201 | NS | 1.70(0.75-3.88) |
|  | C allele | 334 | 81.5 | 1538 | 81.5 | 0.999 | NS | 1.00(0.76-1.32) |
| Positive pathergy reaction |  | 234 |  | 915 |  |  |  |  |
|  | CC | 147 | 62.8 | 606 | 66.2 | 0.327 | NS | 0.86(0.64-1.16) |
|  | CT | 76 | 32.5 | 290 | 31.7 | 0.818 | NS | 1.04(0.76-1.41) |
|  | TT | 11 | 4.7 | 19 | 2.1 | 0.025 | NS | 2.33(1.09-4.96) |
|  | C allele | 370 | 79.1 | 1502 | 82.1 | 0.134 | NS | 0.82(0.64-1.06) |
| Hypopyon |  | 260 |  | 889 |  |  |  |  |
|  | CC | 171 | 65.8 | 582 | 65.5 | 0.928 | NS | 1.01(0.76-1.36) |
|  | CT | 81 | 31.2 | 285 | 32.1 | 0.783 | NS | 0.96(0.71-1.29) |
|  | TT | 8 | 3.0 | 22 | 2.4 | 0.592 | NS | 1.25(0.55-2.84) |
|  | C allele | 423 | 81.3 | 1449 | 81.5 | 0.938 | NS | 0.99(0.77-1.27) |
| NS: not significant; 95 % CI: 95 % confidence interval | | | | | | | | |


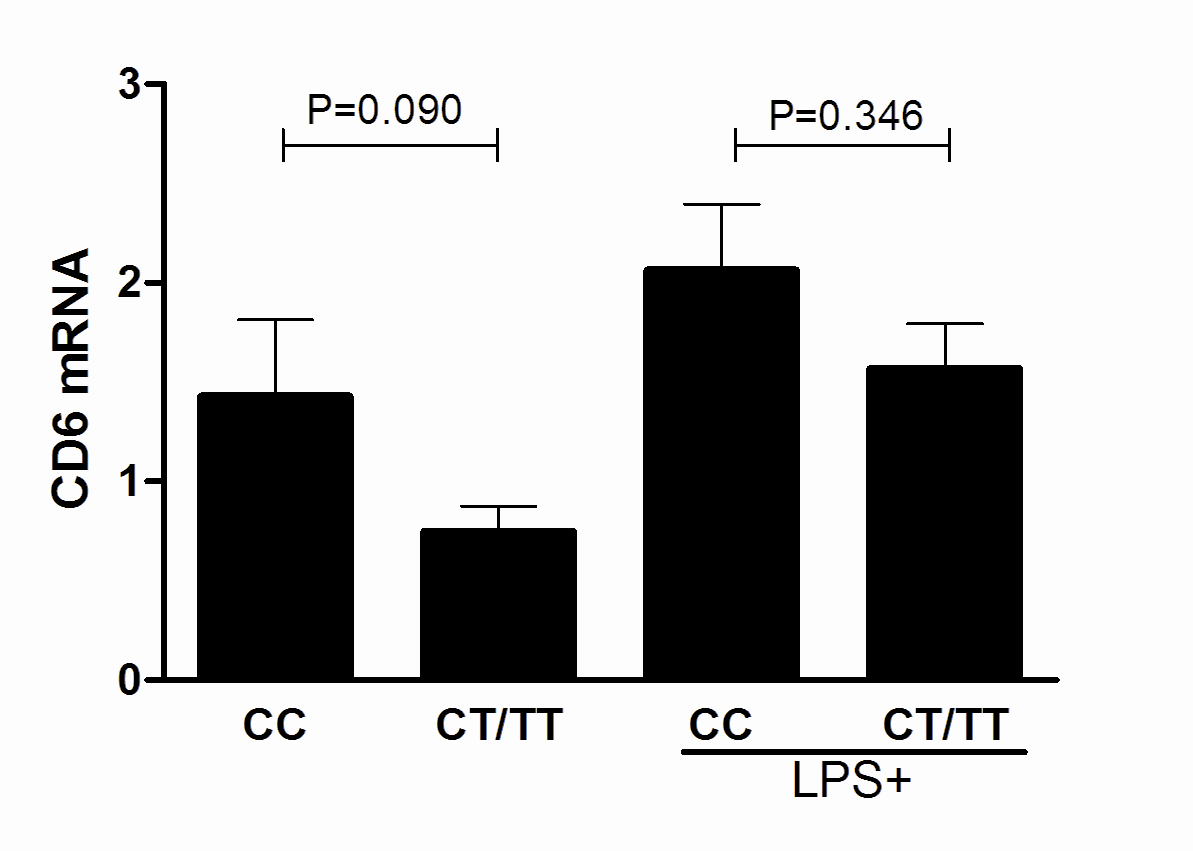


Supplementary Figure.1 The influence of various rs11230563 genotypes on the expression of CD6. CD6 expression in non-stimulated PBMCs and LPS stimulated PBMCs from normal controls carrying different genotypes of rs11230563 (CT/TT=16, CC=16)
